# A Descriptive Study on Sepsis: Causes, Outcomes, and Adherence to Guidelines on Patients with Sepsis at a Tertiary Care Hospital in Sri Lanka

**DOI:** 10.1155/2020/7971387

**Published:** 2020-07-15

**Authors:** Anne Thushara Matthias, J. Indrakumar, Tharushi Ranasinghe, Shalini Wijekoon, Charuni Yashodara

**Affiliations:** University of Sri Jayewardenepura, Sri Lanka

## Abstract

The global incidence of sepsis is increasing, and mortality remains high. The mortality is even higher in resource-poor countries where facilities and equipment are limited. The *Surviving Sepsis Campaign* (*SSC*) recommends an updated hour-1 bundle based on the evidence from the International Guidelines for Management of Sepsis and Septic Shock 2018. To reduce mortality from sepsis, compliance with the “bundle” is essential. Data from developing countries like Sri Lanka on the management of sepsis according to the SSC guidelines are not available. Hence, this study looks at the patient characteristics and management of septic patients at a tertiary care hospital in Sri Lanka. Patients admitted to the University Medical Unit of Colombo South Teaching Hospital from January to August 2019 fulfilling the inclusion criteria were included. The hour-1 sepsis bundle adherence, demographic data, and management were recorded. There were 387 patients: 163 males and 224 females. The age range was 15-95 with a mean age of 63. 83.7% were direct admissions while 16.3% were transfers from a peripheral hospital. The most common source of infection was urine (82 (21.2%)) followed by blood stream (105 (27.1%)) and skin and soft tissue (114 (29.5%)). One-hour SSC bundle compliance is as follows: administration of intravenous fluids: 42 (10.9%), blood cultures before antibiotics: 225 (58.1%), first dose antibiotic: 15 (3.9%), and arterial blood gas: 60 (15.5%). Staffing capacity did not make a difference to adherence to the bundle. The study mortality rate was 37 (9.6%). Binary logistic regression indicates that quick sequential organ failure assessment (qSOFA) score is a significant predictor of mortality (chi‐square = 35.08, df = 3, and *p* = 0.001 (<0.05)) with an odds ratio (OR) of 7.529 (95% CI 3.597-14.323). The other predictors, age, sex, adherence to sepsis care bundle, and comorbidities, were not significant. In conclusion, mortality of sepsis is high and adherence to sepsis care bundle is poor in Sri Lanka even at a tertiary care hospital. Education and training of staff are needed to boost adherence. This will in turn improve quality of care and outcomes of septic patients in resource-poor countries.

## 1. Introduction

Sepsis is defined as an overwhelming and potentially life-threatening inflammatory response of the body to infection resulting in organ dysfunction and death causing significant morbidity and mortality[1]. The definition of sepsis has changed over the years with various criteria used for diagnosis such as surviving sepsis criteria and qSOFA [2].

Sepsis is a major burden on the healthcare system of any country. Sepsis incidence rates are up to 535 cases per 100,000 person-years and rising[3]. In-hospital mortality remains high at 25–30%. The majority of these are in low- and middle-income countries (LMICs) [4]. In a multicentre study, it was revealed that sepsis in Southeast Asia has a high mortality [5]. There is paucity of data regarding sepsis in Sri Lanka.

To fight the battle against sepsis, the Surviving Sepsis Campaign, a collaboration between the European Society of Intensive Care Medicine, the Society of Critical Care Medicine, and the International Sepsis Forum, was formed in October 2002. The campaign issued a consensus statement in 2004, the first Surviving Sepsis Campaign guideline [4]. Over the years, several updates have been done to optimize the diagnosis and management of sepsis. The concept of care bundles was developed so as to improve the compliance to guidelines. Taking into account the current international and local guidelines on sepsis, the four major pillars of sepsis are blood culture, antibiotics, arterial blood gas (ABG), and fluid therapy[6]. In response to the publication of “Surviving Sepsis Campaign: International Guidelines for Management of Sepsis and Septic Shock: 2016 [7],” a revised “hour-1 bundle” was introduced in “The Surviving Sepsis Campaign Bundle: 2018 Update”[8] by the introduction of a one-hour bundle instead of the previously published 3-hour and 6-hour bundles of care, keeping in line with the latest scientific evidence.

Since limited research has been done regarding sepsis in Sri Lanka, this research was conducted as a descriptive study based on the patients with sepsis in a major tertiary care unit in the country. It will provide valuable insight into the characteristics of patients diagnosed with sepsis and adherence to Surviving Sepsis Campaign guidelines, help in evaluating the clinical outcomes of septic patients, and thus improve the quality of care.

## 2. Methodology

### 2.1. Study Design

This study was a descriptive cohort study conducted prospectively at Colombo South Teaching Hospital (CSTH). CSTH is a tertiary care unit situated in the south of Colombo and is the study setting selected for the study. The selected hospital consists of 1100 beds.

In this cross-sectional study, we prospectively recruited patients aged ≥18 years with suspected or documented sepsis fulfilling the qSOFA ≥ 2 criteria as redefined by a task force convened by national societies including the Society of Critical Care Medicine (SCCM) and the European Society of Intensive Care Medicine (ESICM) termed sepsis-3[9] admitted to University Medical Unit, CSTH. The patients not fulfilling the new sepsis criteria, those aged <18, those mentally unsound, and those unwilling to provide consent are excluded from the study.

The study was conducted from January 2019 to June 2019.

All details were gathered from the Bed Head Tickets (BHTs) and recorded in a data sheet by trained research assistants. Patients were contacted at 30 days to review the outcomes.

### 2.2. Definitions

Patient's demographics were defined as age and sex.

*Sepsis*: life-threatening organ dysfunction caused by a dysregulated host response to infection.

*Organ dysfunction*: an acute change in total sequential organ failure assessment (SOFA) scores ≥ 2 points consequent to the infection.

*Clinical criteria to identify septic shock*: sepsis with persisting hypotension requiring vasopressors to maintain MAP ≥ 65 mmHg and having a serum lactate > 2 mmol/L despite adequate volume resuscitation.

*Time zero*: the time of diagnosis of sepsis. This is the time from which 1 h, 3 h, and 6 h were counted to see whether the management of sepsis within this period was done according to the guideline. qSOFA score at the time of diagnosis was measured.

The statistics were assessed by SPSS Version 17. Categorical variables are described using frequencies. Continuous variables are described as mean and standard deviation if normally distributed or median and interquartile range if not. We used logistic regression models to assess the factors associated with mortality.

## 3. Results

### 3.1. Demography

There were 387 patients: 163 males and 224 females. The age range was 15-95 with a mean age of 63.

### 3.2. Admissions

83.7% were direct admissions to CSTH while 16.3% were transfers from a peripheral hospital. The 30-day readmission was 3 (0.7%) and was due to reasons not related to sepsis or the index admission.

### 3.3. Characteristics of Sepsis

The most common source of infection was urine (82 (21.2%)) followed by blood stream (105 (27.1%)) and skin and soft tissue (114 (29.5%)). qSOFA was one in 124 (32%), two in 162 (41.9%), and three in 46 (11.9%).

### 3.4. Surviving Sepsis Bundle Compliance

One-hour SSC bundle compliance is as follows: administration of intravenous fluids: 42 (10.9%), blood cultures before antibiotics: 225 (58.1%), first dose antibiotic: 15 (3.9%), and arterial blood gas: 60 (15.5%). Inotropes were given to 61 (15.8%) patients within the first 6 hours.

Staffing capacity did not make a difference to adherence to the bundle.

### 3.5. Mortality

The study mortality was 37 (9.6%). Binary logistic regression indicates that quick sequential organ failure assessment score (qSOFA) is a significant predictor of mortality (chi‐square = 35.08, df = 3, and *p* = 0.001 (<0.05)) with an odds ratio (OR) of 7.529 (95% CI 3.597-14.323). The other predictors, age, sex, adherence to sepsis care bundle, and comorbidities, were not significant.

## 4. Discussion

The mean age in our study was 63 which was slightly older than the MOSAICS cohort of 59.2 years [10] and was similar to the international cohort with a mean age of 64 years [11]. The comorbidities which were common in the cohort included DM (182 (46.8%)), HPT (135 (34.7)), IHD (107 (27.5)), and CKD (95 (24.4)).

The overall bundle adherence in our study was poor. In a study done in Asia, the compliance to the severe sepsis resuscitation bundle was low, similar to studies in Europe and North America [10, 12] According to many multicentre trials, a survival benefit was seen with adherence to the severe sepsis/septic shock management bundle [10, 13]. Many studies support the efficacy of the intervention bundles in the management of patients with sepsis. One study showed that patients whose care included compliance with all of these metrics had a 40% reduction in the odds of dying in the hospital with the 3 h bundle and 36% for the 6 h bundle [14].

One critical factor in the treatment of sepsis is the administration of antibiotics to target the underlying cause of infection. The choice of antibiotics depends on the source of infection and local antibiotic guidelines. The very poor rate of administration of antibiotics (3.9%) in our study is devastating. Similar studies done to audit the bundle adherence have found higher rates of antibiotic administration such as 76% (344/454) [15]. However, the blood cultures were taken in more than half the population before antibiotics and this indicates that they have understood the need for proper cultures and administration of antibiotics as soon as possible. Therefore, the delay in the administration of antibiotics seems to be the key factor that needs rectifying. This can be achieved through greater emphasis on implementing the SSC 1-hour bundle through educational programs.

Intravenous fluid was given in 10.9%. This is also low compared to other studies (50%) and administration of adrenergic agents in 71% of patients with hypotension (135/191) [16].

The compliance rate for fluids in MOSAICS was 81.4% [10]. MOSAICS was a large trial. One of the difficulties of achieving this target requires early identification of hypotension to initiate fluid challenge. Most patients in our study were diagnosed in the acute general medical wards where intensive hourly monitoring of blood pressure was unfeasible.

In our study, 69 (17.7%) had low BP, and out of them, inotropes have been given to 61 (15.1%). This is good compliance with the guideline. The timing of administration of inotropes has to improve.

The study mortality rate was 37%. The hospital mortality rate of the study was 26.8% in a cohort of ICU admissions in Hong Kong, and in the large MOSAICS trial, it was 44.5%. In a multicentre trial done in Asia, the mortality rate was 13% but the bundle adherence in this study was not studied. In a large study by Lie et al. [15], the difference in mortality is multifactorial. The principal difference for the mortality to be high could be attributed to poor administration of antibiotics early[10].

### 4.1. Limitations and Generalisability

The limitation of this study includes the small sample size. As it was done over a period of few months at a tertiary care unit, the results can be generalised to a large degree.

## 5. Conclusions

The adherence to Surviving Sepsis Campaign bundles needs to improve in our setting. This would in turn reduce mortality associated with sepsis.

## Data Availability

The data used to support the findings of this study are available from the corresponding author upon request.
